# Religio Medici Modereni

**Published:** 1915-08-21

**Authors:** Neo Kama


### THE EDITOR'S LETTER-BOX.

### "Religio Medici Moderenì."

*To the Editor of* The Hospital.

Sir,—I am sorry you should have wasted so much of your valuable space on a criticism on this little book of mine, though why your critic should have tacked on the *sic* I fail to grasp, as *moderenus* is a good Latin adjective. I have the greatest regard for the female sex, nay love, but not for female logic, especially for the logic of the suffragette. The logic of your criticism writer is female and of the suffragette brand. The critic writes of me, "He does not suggest that men should be deprived of votes because of their incapacity to bear children." Quite true. I do not, never did, nor intend to. Again, "To hear that 'the man must be either a madman or a fool who asserts that there is no continuity after death' is rather astonishing from the point of view of one who dismissed Christian dogmas on the ground that they were irrational." What I try to show is that rationally there is possibly a continuity not of self, but of our essence after death. The professors of dogmatic Christianity admit they hold their dogmas by faith alone, and admit their beliefs to be irrational from a rationalist's point of view. Again she remarks, "The author will probably remain the only member of his Church." I emphatically stated I hold with no Churches nor dogmas, though it is extremely hard to keep clear of the latter, which may be amusing.

There is an old saying that a woman in writing places the real object of her effort in a postscript. Your critic unfortunately omits the postscript. There are eighteen short chapters in my little book, and fourteen deal more or less with the relations of what I call "Bion," yet there is not a word in her two half-columns which refers to this, my principal thesis.

The remarks about Kropotkin's notion and mine of patriotism I will not waste your space by discussing, except to state that personally I have never met a *real* man who wished to merge his own race into a cosmopolitan conglomerate. The only nation who is strenuously striving to make a universal nation of mankind with a common "Kultur" at present is the accurst Germans.

What I have written in the book is not food for babes, nor for young maidens nor old maids, but for strong stomachs. Much of it is wrong possibly, but it is not a subject for illogical piffle but for logical hammer-blows. Some of my notions are entirely new as far as I know, and may be entirely wrong. This I admit.

The only other criticism of my little work that I have so far seen is extremely short, and appears in the *Times* (Literary Supplement) of July 22. It is: "There are many acute observations in these chapters on life and its meaning by a thoughtful, scientific mind which is entirely antipathetic to any kind of dogma, and has a theory of a living essence in the Universe to which he gives the name of 'Bion,' somewhat akin to what he describes as 'the old discarded theory of vitalism.'"—Yours, etc.,

"Neo Kama."

[As the writer of the review referred to was a man our correspondent's letter loses some of its point.—Ed. The Hospital.]

["Modereni" will hardly pass. The author, naturally perhaps, seems to have sacrificed accuracy to alliteration. "Moderni," though Low Latin, is Latin. "Modereni" makes one write "*sic.*" "Hodierni" would probably have been preferred by Cicero But this perhaps is a trifle. I wish I had space to deal with the curious confusion of the author's ideas. This confusion can be shown shortly, from the fact of his quoting with approval from another review the remark that a scientific mind is entirely antipathetic to all dogma. A dogma, literally "an opinion," is a principle or tenet (like Euclid's postulates), just as a law of Nature is a working hypothesis, or, in the jargon of the schools, an induction from constantly recurring phenomena, whereby we are enabled to state that the same conditions always accompany the same results. In short, the laws of Nature are the dogmas of science; and "science" without dogmas, as this volume shows, becomes a chaos of observations, deductions, and loose thinking. For observations are as useless as *a priori* guesses unless and until there is some principle or dogma by which to test them. Neo Kama makes the old mistake of thinking that you can have one without the other. Not so the Newtons of science, nor the great theologians, who are thus shown to be the better reasoners and the better scientists respectively, possessing withal that saving scientific grace—a sense of humour. The work is an extraordinary patchwork. To see it at its best read the last paragraph of the chapter on "Rewards" (p. 110). Who would venture to improve it? But it makes mincemeat of almost all of the rest of the chapter. Let me rather dwell on such paragraphs as the best however.—Your Reviewer.]

### Heating of Leicester Infirmary.

*To the Editor of* The Hospital.

